# Supplementary material for: Modeling glioblastoma heterogeneity as a dynamic network of cell states
Source: Mol Syst Biol. 2021 Sep 16;17(9):e10105. doi: 10.15252/msb.202010105 (PMC8444284; doi:10.15252/msb.202010105)
Supplement: Supplementary file 6 — Source Data for Figure 5 [file MSB-17-e10105-s004.zip › Figure5A_sourcedata/GSEA_3017/nosigdb_stateA.GseaPreranked.1622795026485/RICHARDS_CAHOY_JNEUROSCI_2008_ASTROCYTE.html]

Details for gene set RICHARDS\_CAHOY\_JNEUROSCI\_2008\_ASTROCYTE[GSEA]

|  || Dataset | state53017 |
| Phenotype | NoPhenotypeAvailable |
| Upregulated in class | na\_neg |
| GeneSet | RICHARDS\_CAHOY\_JNEUROSCI\_2008\_ASTROCYTE |
| Enrichment Score (ES) | -0.38120458 |
| Normalized Enrichment Score (NES) | -2.6949008 |
| Nominal p-value | 0.0 |
| FDR q-value | 0.0 |
| FWER p-Value | 0.0 |
Table: GSEA Results Summary

  

Fig 1: Enrichment plot: RICHARDS\_CAHOY\_JNEUROSCI\_2008\_ASTROCYTE      
 Profile of the Running ES Score & Positions of GeneSet Members on the Rank Ordered List

  

| PROBE | GENE SYMBOL | GENE\_TITLE | RANK IN GENE LIST | RANK METRIC SCORE | RUNNING ES | CORE ENRICHMENT || 1 | FABP5 |  |  | 0 | 1.759 | 0.0193 | No |
| 2 | KIF11 |  |  | 6 | 0.870 | 0.0225 | No |
| 3 | PCDH10 |  |  | 14 | 0.734 | 0.0217 | No |
| 4 | DBI |  |  | 17 | 0.683 | 0.0267 | No |
| 5 | ASPM |  |  | 27 | 0.639 | 0.0223 | No |
| 6 | HEY2 |  |  | 33 | 0.617 | 0.0228 | No |
| 7 | CCNB1 |  |  | 37 | 0.599 | 0.0255 | No |
| 8 | VIM |  |  | 46 | 0.543 | 0.0214 | No |
| 9 | TPX2 |  |  | 66 | 0.488 | 0.0027 | No |
| 10 | ECHS1 |  |  | 67 | 0.487 | 0.0081 | No |
| 11 | FGFBP3 |  |  | 75 | 0.477 | 0.0045 | No |
| 12 | CTSC |  |  | 96 | 0.456 | -0.0158 | No |
| 13 | GHITM |  |  | 103 | 0.450 | -0.0184 | No |
| 14 | ZEB1 |  |  | 109 | 0.446 | -0.0199 | No |
| 15 | CTBP2 |  |  | 142 | 0.413 | -0.0558 | No |
| 16 | S100A4 |  |  | 148 | 0.409 | -0.0576 | No |
| 17 | S100A16 |  |  | 156 | 0.404 | -0.0620 | No |
| 18 | EIF4EBP2 |  |  | 158 | 0.403 | -0.0588 | No |
| 19 | TNC |  |  | 159 | 0.402 | -0.0544 | No |
| 20 | ARHGAP11A |  |  | 162 | 0.399 | -0.0526 | No |
| 21 | KIF2C |  |  | 165 | 0.397 | -0.0508 | No |
| 22 | MAP3K1 |  |  | 167 | 0.396 | -0.0477 | No |
| 23 | MYC |  |  | 169 | 0.395 | -0.0446 | No |
| 24 | CCDC6 |  |  | 172 | 0.394 | -0.0428 | No |
| 25 | MRPS6 |  |  | 181 | 0.389 | -0.0487 | No |
| 26 | CKLF |  |  | 183 | 0.388 | -0.0457 | No |
| 27 | METRN |  |  | 186 | 0.387 | -0.0440 | No |
| 28 | SERPINE2 |  |  | 201 | 0.373 | -0.0576 | No |
| 29 | SMC4 |  |  | 217 | 0.367 | -0.0725 | No |
| 30 | RAB13 |  |  | 220 | 0.366 | -0.0710 | No |
| 31 | UGDH |  |  | 224 | 0.363 | -0.0708 | No |
| 32 | AMOT |  |  | 242 | 0.353 | -0.0884 | No |
| 33 | ALCAM |  |  | 252 | 0.348 | -0.0959 | No |
| 34 | SLC25A5 |  |  | 266 | 0.340 | -0.1086 | No |
| 35 | BCAN |  |  | 276 | 0.334 | -0.1163 | No |
| 36 | SEPT9 |  |  | 284 | 0.331 | -0.1215 | No |
| 37 | MT1X |  |  | 285 | 0.331 | -0.1179 | No |
| 38 | IDE |  |  | 299 | 0.326 | -0.1308 | No |
| 39 | SWAP70 |  |  | 323 | 0.317 | -0.1563 | No |
| 40 | FARP1 |  |  | 332 | 0.315 | -0.1630 | No |
| 41 | CLIC1 |  |  | 342 | 0.311 | -0.1709 | No |
| 42 | PRC1 |  |  | 350 | 0.310 | -0.1764 | No |
| 43 | SGPL1 |  |  | 360 | 0.306 | -0.1844 | No |
| 44 | SLC20A1 |  |  | 361 | 0.306 | -0.1810 | No |
| 45 | TTF2 |  |  | 367 | 0.305 | -0.1840 | No |
| 46 | RRM2 |  |  | 383 | 0.300 | -0.1997 | No |
| 47 | TXNDC5 |  |  | 406 | 0.291 | -0.2242 | No |
| 48 | ATP13A4 |  |  | 410 | 0.290 | -0.2249 | No |
| 49 | P4HA1 |  |  | 411 | 0.290 | -0.2217 | No |
| 50 | NFIA |  |  | 413 | 0.289 | -0.2198 | No |
| 51 | DOCK1 |  |  | 414 | 0.289 | -0.2166 | No |
| 52 | ACBD5 |  |  | 422 | 0.288 | -0.2223 | No |
| 53 | HMGB2 |  |  | 424 | 0.288 | -0.2204 | No |
| 54 | NUSAP1 |  |  | 430 | 0.286 | -0.2236 | No |
| 55 | NUF2 |  |  | 434 | 0.285 | -0.2242 | No |
| 56 | USP54 |  |  | 438 | 0.285 | -0.2249 | No |
| 57 | NCAPD2 |  |  | 440 | 0.283 | -0.2231 | No |
| 58 | ENTPD1 |  |  | 444 | 0.282 | -0.2238 | No |
| 59 | CACNG8 |  |  | 448 | 0.281 | -0.2245 | No |
| 60 | ANP32E |  |  | 453 | 0.280 | -0.2264 | No |
| 61 | ABI1 |  |  | 459 | 0.279 | -0.2297 | No |
| 62 | CUTC |  |  | 484 | 0.273 | -0.2570 | No |
| 63 | VTI1A |  |  | 489 | 0.272 | -0.2591 | No |
| 64 | PSAT1 |  |  | 491 | 0.271 | -0.2574 | No |
| 65 | EPC1 |  |  | 501 | 0.270 | -0.2658 | No |
| 66 | NUP93 |  |  | 506 | 0.269 | -0.2679 | No |
| 67 | PLEKHO1 |  |  | 511 | 0.269 | -0.2700 | No |
| 68 | PXDN |  |  | 524 | 0.266 | -0.2822 | No |
| 69 | TBC1D12 |  |  | 529 | 0.264 | -0.2844 | No |
| 70 | ZFP36L1 |  |  | 532 | 0.264 | -0.2840 | No |
| 71 | ITGA6 |  |  | 544 | 0.261 | -0.2950 | No |
| 72 | VEGFA |  |  | 546 | 0.261 | -0.2934 | No |
| 73 | FZD2 |  |  | 551 | 0.259 | -0.2957 | No |
| 74 | SUPV3L1 |  |  | 570 | 0.256 | -0.3156 | No |
| 75 | PTEN |  |  | 572 | 0.255 | -0.3140 | No |
| 76 | SOX9 |  |  | 577 | 0.254 | -0.3163 | No |
| 77 | TMPO |  |  | 584 | 0.253 | -0.3211 | No |
| 78 | AGT |  |  | 587 | 0.252 | -0.3209 | No |
| 79 | TIMM8A |  |  | 589 | 0.252 | -0.3194 | No |
| 80 | ITGAV |  |  | 599 | -0.250 | -0.3280 | No |
| 81 | NR3C1 |  |  | 610 | -0.256 | -0.3378 | No |
| 82 | PHKB |  |  | 616 | -0.259 | -0.3413 | No |
| 83 | ABAT |  |  | 617 | -0.259 | -0.3385 | No |
| 84 | DAG1 |  |  | 622 | -0.260 | -0.3407 | No |
| 85 | GPR37L1 |  |  | 624 | -0.261 | -0.3391 | No |
| 86 | KCNJ16 |  |  | 634 | -0.264 | -0.3475 | No |
| 87 | GNG12 |  |  | 640 | -0.265 | -0.3509 | No |
| 88 | MAGI1 |  |  | 642 | -0.266 | -0.3493 | No |
| 89 | PTPRA |  |  | 643 | -0.267 | -0.3464 | No |
| 90 | ABCA1 |  |  | 647 | -0.268 | -0.3472 | No |
| 91 | SOCS3 |  |  | 651 | -0.270 | -0.3480 | No |
| 92 | CHST2 |  |  | 652 | -0.271 | -0.3451 | No |
| 93 | NOTCH1 |  |  | 666 | -0.280 | -0.3584 | No |
| 94 | SPRY2 |  |  | 669 | -0.282 | -0.3579 | No |
| 95 | CHD9 |  |  | 673 | -0.284 | -0.3585 | No |
| 96 | CKB |  |  | 676 | -0.287 | -0.3579 | No |
| 97 | PTTG1IP |  |  | 678 | -0.290 | -0.3560 | No |
| 98 | RHOJ |  |  | 682 | -0.293 | -0.3566 | No |
| 99 | EFEMP2 |  |  | 686 | -0.294 | -0.3571 | No |
| 100 | GPC4 |  |  | 687 | -0.295 | -0.3539 | No |
| 101 | KCNIP1 |  |  | 689 | -0.295 | -0.3519 | No |
| 102 | MDFIC |  |  | 709 | -0.308 | -0.3726 | No |
| 103 | TSC22D4 |  |  | 716 | -0.312 | -0.3767 | No |
| 104 | S100A6 |  |  | 717 | -0.313 | -0.3733 | No |
| 105 | FZD7 |  |  | 721 | -0.316 | -0.3736 | No |
| 106 | ZEB2 |  |  | 722 | -0.319 | -0.3701 | No |
| 107 | EMP1 |  |  | 724 | -0.322 | -0.3679 | No |
| 108 | ID2 |  |  | 727 | -0.323 | -0.3668 | No |
| 109 | ELOVL2 |  |  | 735 | -0.329 | -0.3721 | No |
| 110 | HES1 |  |  | 736 | -0.331 | -0.3684 | No |
| 111 | PON2 |  |  | 743 | -0.338 | -0.3723 | No |
| 112 | MYO6 |  |  | 750 | -0.343 | -0.3761 | No |
| 113 | HEY1 |  |  | 751 | -0.344 | -0.3724 | No |
| 114 | SRGAP3 |  |  | 755 | -0.345 | -0.3724 | No |
| 115 | SLC4A4 |  |  | 763 | -0.355 | -0.3773 | Yes |
| 116 | BOC |  |  | 766 | -0.359 | -0.3759 | Yes |
| 117 | MSI2 |  |  | 771 | -0.361 | -0.3770 | Yes |
| 118 | ITGA7 |  |  | 773 | -0.362 | -0.3743 | Yes |
| 119 | ITGB8 |  |  | 774 | -0.363 | -0.3703 | Yes |
| 120 | SPARC |  |  | 782 | -0.369 | -0.3751 | Yes |
| 121 | MLC1 |  |  | 783 | -0.369 | -0.3711 | Yes |
| 122 | MACF1 |  |  | 785 | -0.371 | -0.3683 | Yes |
| 123 | GNAO1 |  |  | 788 | -0.373 | -0.3667 | Yes |
| 124 | CDH4 |  |  | 794 | -0.376 | -0.3689 | Yes |
| 125 | SQSTM1 |  |  | 795 | -0.379 | -0.3647 | Yes |
| 126 | SEC14L1 |  |  | 807 | -0.388 | -0.3744 | Yes |
| 127 | PHPT1 |  |  | 811 | -0.393 | -0.3739 | Yes |
| 128 | FHL1 |  |  | 814 | -0.397 | -0.3720 | Yes |
| 129 | LTBP1 |  |  | 815 | -0.397 | -0.3677 | Yes |
| 130 | NUDT3 |  |  | 817 | -0.399 | -0.3646 | Yes |
| 131 | C1QTNF6 |  |  | 819 | -0.401 | -0.3614 | Yes |
| 132 | IGFBP2 |  |  | 822 | -0.404 | -0.3595 | Yes |
| 133 | GJA1 |  |  | 823 | -0.405 | -0.3551 | Yes |
| 134 | DDAH1 |  |  | 828 | -0.413 | -0.3556 | Yes |
| 135 | PRRX1 |  |  | 829 | -0.413 | -0.3511 | Yes |
| 136 | RORB |  |  | 830 | -0.413 | -0.3466 | Yes |
| 137 | TTYH3 |  |  | 834 | -0.428 | -0.3457 | Yes |
| 138 | ATF3 |  |  | 836 | -0.428 | -0.3422 | Yes |
| 139 | PLOD2 |  |  | 838 | -0.431 | -0.3388 | Yes |
| 140 | TGFB2 |  |  | 840 | -0.432 | -0.3353 | Yes |
| 141 | POU3F2 |  |  | 843 | -0.437 | -0.3330 | Yes |
| 142 | CHPT1 |  |  | 844 | -0.437 | -0.3282 | Yes |
| 143 | SDCBP |  |  | 845 | -0.438 | -0.3234 | Yes |
| 144 | ZFP36L2 |  |  | 848 | -0.443 | -0.3211 | Yes |
| 145 | ELN |  |  | 852 | -0.445 | -0.3200 | Yes |
| 146 | TAX1BP1 |  |  | 854 | -0.447 | -0.3164 | Yes |
| 147 | TMEM47 |  |  | 855 | -0.447 | -0.3115 | Yes |
| 148 | CPEB4 |  |  | 857 | -0.447 | -0.3079 | Yes |
| 149 | SLC1A2 |  |  | 860 | -0.450 | -0.3054 | Yes |
| 150 | IFITM2 |  |  | 864 | -0.460 | -0.3042 | Yes |
| 151 | UPP1 |  |  | 867 | -0.467 | -0.3016 | Yes |
| 152 | ANXA2 |  |  | 872 | -0.482 | -0.3014 | Yes |
| 153 | ZNF106 |  |  | 873 | -0.483 | -0.2961 | Yes |
| 154 | RGS12 |  |  | 880 | -0.504 | -0.2981 | Yes |
| 155 | PDGFC |  |  | 883 | -0.515 | -0.2950 | Yes |
| 156 | JUN |  |  | 886 | -0.525 | -0.2918 | Yes |
| 157 | F3 |  |  | 888 | -0.526 | -0.2873 | Yes |
| 158 | PDPN |  |  | 890 | -0.529 | -0.2827 | Yes |
| 159 | GPC6 |  |  | 896 | -0.540 | -0.2831 | Yes |
| 160 | GPM6B |  |  | 897 | -0.542 | -0.2772 | Yes |
| 161 | A2M |  |  | 898 | -0.545 | -0.2712 | Yes |
| 162 | ITGB5 |  |  | 903 | -0.558 | -0.2702 | Yes |
| 163 | ATP1B2 |  |  | 904 | -0.558 | -0.2640 | Yes |
| 164 | MT3 |  |  | 905 | -0.559 | -0.2579 | Yes |
| 165 | SDC2 |  |  | 908 | -0.568 | -0.2542 | Yes |
| 166 | SAT1 |  |  | 909 | -0.573 | -0.2479 | Yes |
| 167 | DKK3 |  |  | 912 | -0.579 | -0.2441 | Yes |
| 168 | CADM1 |  |  | 914 | -0.582 | -0.2390 | Yes |
| 169 | PTX3 |  |  | 919 | -0.603 | -0.2375 | Yes |
| 170 | GLUL |  |  | 920 | -0.612 | -0.2307 | Yes |
| 171 | NEDD9 |  |  | 923 | -0.625 | -0.2264 | Yes |
| 172 | ID1 |  |  | 931 | -0.651 | -0.2281 | Yes |
| 173 | TTYH1 |  |  | 932 | -0.652 | -0.2210 | Yes |
| 174 | CST3 |  |  | 933 | -0.680 | -0.2135 | Yes |
| 175 | CLU |  |  | 938 | -0.685 | -0.2111 | Yes |
| 176 | CEBPD |  |  | 939 | -0.690 | -0.2035 | Yes |
| 177 | NFIX |  |  | 940 | -0.692 | -0.1959 | Yes |
| 178 | HEPACAM |  |  | 943 | -0.709 | -0.1907 | Yes |
| 179 | RNF180 |  |  | 945 | -0.725 | -0.1840 | Yes |
| 180 | CDH2 |  |  | 947 | -0.762 | -0.1769 | Yes |
| 181 | GLIS3 |  |  | 948 | -0.767 | -0.1685 | Yes |
| 182 | GDF15 |  |  | 952 | -0.790 | -0.1636 | Yes |
| 183 | IFITM3 |  |  | 954 | -0.830 | -0.1558 | Yes |
| 184 | CD44 |  |  | 957 | -0.851 | -0.1490 | Yes |
| 185 | COL5A2 |  |  | 958 | -0.856 | -0.1396 | Yes |
| 186 | CNN3 |  |  | 961 | -0.883 | -0.1325 | Yes |
| 187 | ZBTB20 |  |  | 964 | -0.895 | -0.1252 | Yes |
| 188 | PBX1 |  |  | 968 | -0.964 | -0.1184 | Yes |
| 189 | FABP7 |  |  | 971 | -0.994 | -0.1100 | Yes |
| 190 | SLC1A3 |  |  | 973 | -1.097 | -0.0993 | Yes |
| 191 | CYR61 |  |  | 978 | -1.296 | -0.0901 | Yes |
| 192 | ID3 |  |  | 981 | -1.505 | -0.0762 | Yes |
| 193 | PMP22 |  |  | 982 | -1.519 | -0.0595 | Yes |
| 194 | IGFBP5 |  |  | 983 | -1.595 | -0.0420 | Yes |
| 195 | SPARCL1 |  |  | 984 | -1.810 | -0.0222 | Yes |
| 196 | GFAP |  |  | 987 | -2.255 | 0.0000 | Yes |
Table: GSEA details [plain text format]

  

Fig 2: RICHARDS\_CAHOY\_JNEUROSCI\_2008\_ASTROCYTE: Random ES distribution      
 Gene set null distribution of ES for **RICHARDS\_CAHOY\_JNEUROSCI\_2008\_ASTROCYTE**
